# An electron microscopic study of the archaeal feast/famine regulatory protein: 2. Crystal formation by a full length FFRP, pot0434017 (FL11)

**Published:** 2004-01-01

**Authors:** Sanae A. Ishijima, Lester Clowney, Hideaki Koike, Masashi Suzuki

**Affiliations:** *) National Institute of Advanced Industrial Science and Technology (AIST), AIST Tsukuba Center 6-10, 1-1-1, Higashi, Tsukuba, Ibaraki 305-8566, Japan; **) Japan Science and Technology Agency (JST), Core Research for Evolutionary Science and Technology (CREST), Honmachi, 4-1-18, Kawaguchi Center Building, Kawaguchi, Saitama 332-0012, Japan

**Keywords:** Archaea, cryo-electron microscopy, FFRP, hyper-thermophile, transcription factor

## Abstract

In the crystal the archaeal feast/famine regulatory protein pot0434017 (FL11) forms helical cylinders, each extending along the *c* axis and densely packed with a hexagonal symmetry in the *a*/*b* plane. By applying cryo-electron microscopy to protein solutions yielding crystals, with selectively focussing zero-loss electrons accelerated at 200 KV, two types of regular objects were observed, hexagonal (i.e. projections of crystals to their *a*/*b* planes) or rectangular (i.e. projections onto planes perpendicular to *a*/*b*). The two types of images are different in the ranges of sizes, suggesting that the crystallization might initiate by forming a hexagonal sheet on the *a*/*b* plane, subsequently extending along the *c* axis. Some other images obtained were intermediate between regular and amorphous, suggesting that some crystals were growing inside amorphous precipitates by rearranging the protein molecules, and that some larger crystals were growing by absorbing smaller amorphous precipitates. Tubes running parallel to each other were also observed in pieces of thin films. These tubes have hollows in their centers, and their lateral arrangement with a periodicity of ~150 Å and the presence of a helical component ~50 Å suggest that they are projections of the helical cylinders, forming mono-layers on the *a*/*c* or *b*/*c* planes.

## Introduction

The classification, feast/famine regulatory proteins (FFRPs), encompasses archaeal DNA-binding proteins with *E. coli* transcription factors, Lrp (the leucine-responsive regulatory protein) and AsnC (the asparagine synthase *C* gene product).1)–7) The hyper-thermophilic archaeon *Pyrococcus* sp. OT3 has 11 full-length (fl) and 3 demi-FFRPs.2) A fl-FFRP has two domains, an N-terminal DNA-binding domain, and another one C-terminal. Lacking the DNA-binding domain, a demi-FFRP corresponds to the C-domain only of a fl-FFRP.

Using the domains common among the two types, each pair of FFRP monomers fold into a dimer, and by further assembling these dimers higher-order structures are made, i.e. disks composed of four dimers each, and helical cylinders with each six dimers forming a turn. Assembling of a demi-FFRP from *P*. OT3, pot1216151 (DM1) has been studied in amorphous ice using cryo-electron microscopy (EM),7) and in crystal by analyzing X-ray diffraction.8) In these conditions this protein forms disks.

In this paper, the process of forming crystals by a fl-FFRP from *P*. OT3, pot0434017 (FL11), is studied by cryo-EM. This protein transits between assembly forms (Koike, H. *et al*. submitted). In solution, pot0434017 predominantly behaves as dimers, but upon binding a promoter DNA, it forms disks: observations using cryo-EM. While, when crystallized, it assembles into helical cylinders, which continue from one end of crystals to the other end.4)

## Crystallization of pot0434017

Nucleotides coding a histidine-tag were added upstream of gene *pot0434017*, and the modified gene was introduced into the *E. coli* strain BL21 (DE3) using the pET28a plasmid. The expressed protein was purified, after heat treatment at 75°C for 30 min, by affinity chromatography using a Ni-NTA column (Pharmacia) and a 20–1000 mM gradient of imidazole, followed by gel filtration using Sephacryl S-300 (Pharmacia). The protein was concentrated using ultra-filtration to ~20 mg/ml in 50 mM Na-phosphate buffer (pH = 8.0) containing 300 mM NaCl.

**Fig. 1:**
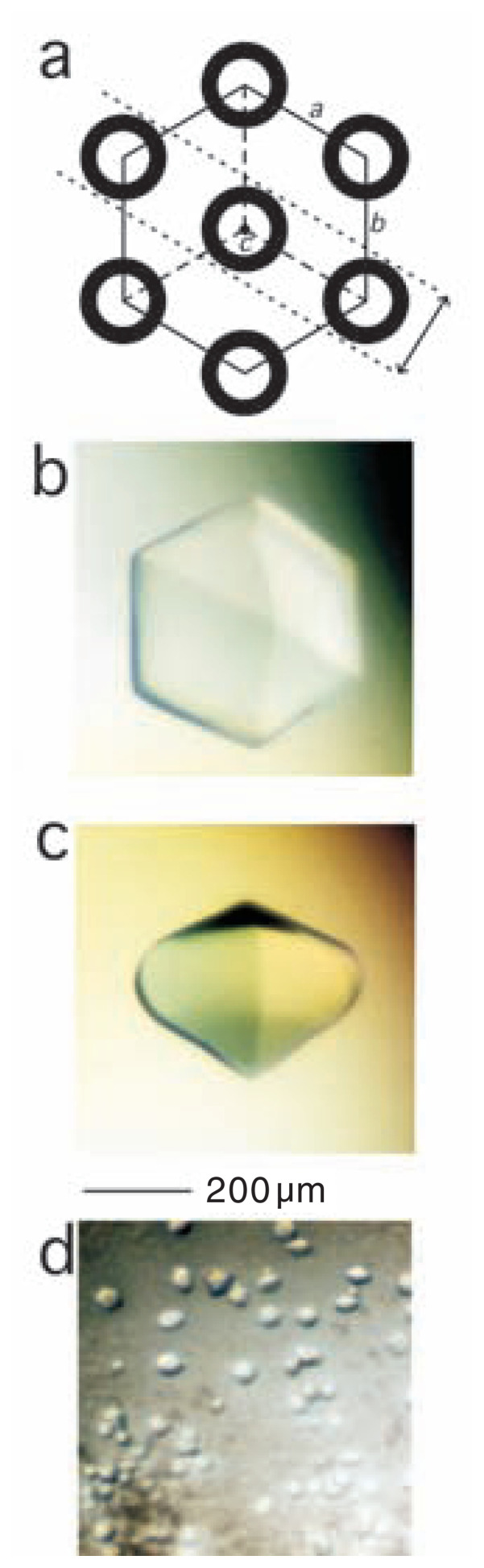
Crystals of pot0434017 observed using an optical microscope. (a) The arrangement of helical cylinders of pot0434017 (circles) in crystals. The unit lengths along the axes, *a*, *b*, *c*, are 130.9 Å, 130.9 Å, and 47.4 Å, respectively. The *c* axis is perpendicular to *a* and *b*. The width of a layer corresponding to those shown in Fig. 4 is indicated by an arrow. (b) and (c) Top (b) and side (c) views of the two largest crystals, photographed using an optical microscope (a binocular, SZ-ST, Olympus, equipped with a polarizer). (d) A solution used for cryo-EM, containing smaller crystals. Note that (b)-(d) are shown at the same scale.

Crystals were obtained by vapor diffusion using the sitting drop method at 20°C: 4 μl of the protein solution was mixed with the same volume of 0.1 M Tris-HCl buffer (pH = 8.5) containing 1.5 M ammonium sulfate and 12% (v/v) glycerol. Usually in ~3 days, crystals ([~0.4 mm] × [~0.4 mm] × [~0.2 mm]) large enough to be used for X-ray diffraction measurements were formed in some wells (top, Fig. 1b, and side, Fig. 1c, views). Since these are too large for EM, solutions yielding smaller crystals in some other wells were studied (Fig. 1d), by selecting the smallest ones optimal for EM.

## Cryo-electron microscopy

In general, by keeping proteins in a frozen-hydrated state, damages caused by electron irradiation can be minimized, i.e. cryo-EM.9),10) This method was further improved, with the discovery that formation of ice crystals, which is harmful to proteins, can be prevented by rapid cooling of the protein solutions.11),12) Thus, each protein solution, 4 μl, was placed on a holey carbon-coated 300 mesh grid (Electron Microscopy Sciences Co.), and frozen in liquid ethane into an amorphous ice state,13),14) using a freezing apparatus (EM CPC, Leica). Here no heavy metal was added, which is often used for enhancing the electron scattering (i.e. positive or negative electron staining). The grid was maintained at a near liquid nitrogen temperature by a holder (CT3500, Oxford), while an electron microscope (Tecnai F20, FEI) was operated at 200 KeV. Zero-loss electrons, selected by an energy filter (GIF200, Gatan), were focused and recorded using a CCD camera (794IF, Gatan). Slight defocusing by several micrometers was used to enhance the contrast.15),16)

## The symmetry inside the crystal

By an X-ray analysis, the space group of pot0434017 (FL11) crystals has been determined to be *P*6_1_224); the unit cell lengths are 130.9 Å(*a*)/130.9 Å(*b*)/47.4 Å(*c*). In each crystal the *a* and *b* axes are identical, while the *c* axis is perpendicular to them (Fig. 1a). Helical cylinders formed by pot0434017 run parallel to this *c* axis. While, looked along the *c* axis to the *a*/*b* plane, these cylinders are densely packed with a hexagonal symmetry (Fig. 1a). Inside a cylinder, each helical turn is formed by six consecutive dimers, spanning 47.4 Å.

In short, this formation resembles a bundle of pencils, densely packed with no space made between their hexagonal cross-sections. However, to represent the crystal more accurately, each pencil needs to be twisted by adopting a helicity. Also the lead inside the pencil needs to be removed in order to create the empty space in the center. In the crystal cylinder, such empty space formed is possibly used for ligands to diffuse along.1)

## Hexagonal and rectangular images

**Fig. 2:**
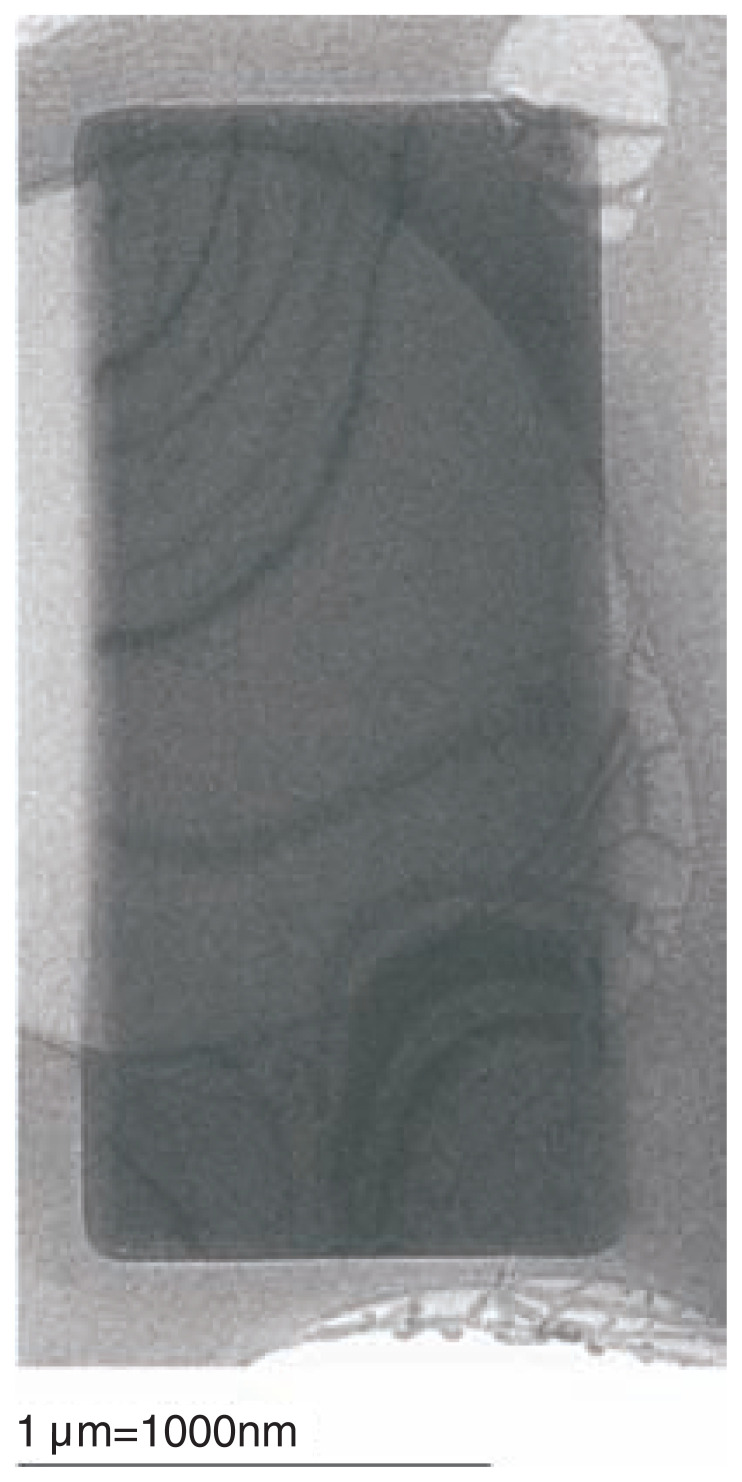
Another crystal of pot0434017 observed using cryo-EM (the original magnification 7,500).

**Fig. 3:**
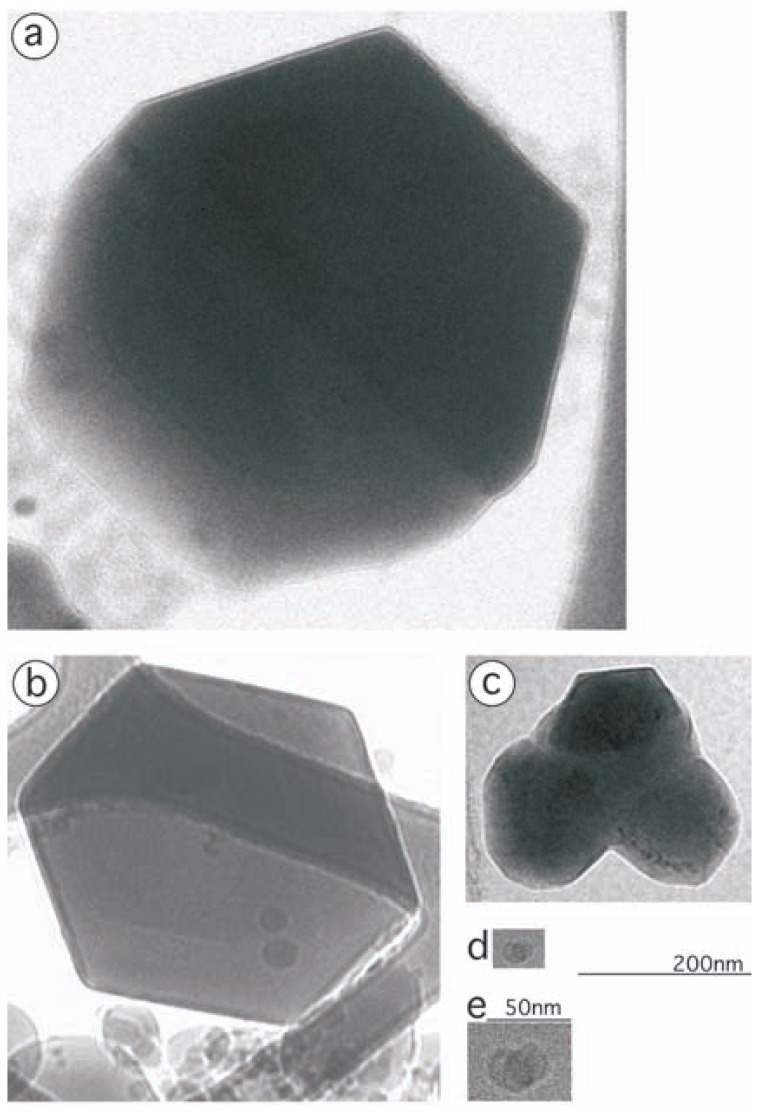
A crystal (b) and pseudo-crystals (a, c, d, e) of pot0434017 observed using cryo-EM. (a)-(d) are shown at the same scale (see the scale 200 nm), while (e) is an enlargement of (d) (see the scale 50 nm). The original magnifications are 27,000 (a, c), 40,000 (b) and 105,000 (d, e), respectively.

In the electron micrographs, two types of regularly shaped images were found with similar frequencies; hexagons with the diameters of 40–700 nm, i.e. 0.04–0.7 μm (Fig. 3b) and rectangles, 0.04 × 0.16 μm, 0.2 × 0.2 μm, 0.6 × 0.7 μm, (0.1–0.6) × (1.4–2.9) μm, and 2.3 × 4.8 μm (Fig. 2). Here some of the latter are significantly larger than the former.

Obviously, the hexagons are the views of each crystal projected along the *c* axis to the *a*/*b* plane. The orientation of crystals observed, when measuring their X-ray diffraction, is consistent with this conclusion. Also, electron diffraction in hexagonal symmetries was observed from the hexagons, showing that internal and external symmetries coincide (data not shown).

The rectangular images (Fig. 2), on the other hand, must be presenting projections with another orientation of crystals, most likely perpendicular to the one resulting in the hexagons: the *c* axis runs inside each rectangular plane.

One might imagine that the *c* axis is placed parallel to the pair of longer sides of each rectangle, since cylinders are long but narrow. However, this is unlikely to be the case. In crystals large enough for observation using an optical microscope (Fig. 1c), the diameters across the hexagonal equators (i.e. the maximum lengths on the *a*/*b* planes) are larger than the inter-polar distances (i.e. the maximum lengths in the *c* directions). Thus, as a natural extension of this observation, it is more likely that in each rectangle observed by EM the *c* axis runs along the pair of shorter sides.

While these rectangles are quite regular, larger crystals observed by optical microscopy are “double-pyramid”-like, having edges at the two ends (Fig. 1c): the length of each cylinder decreases, while shifting on the equatorial plane from the center to periphery. Apparently, crystals become more globular, while growing larger. A sphere is the most stable structure, in terms of the tightest internal interaction, combined with the smallest surface exposed. To maintain an architecture with increasing the size, adopting a more globular structure appears to be an appropriate tactic.

In general, the larger the sizes of hexagons or rectangles, the higher the scattering densities (i.e. less transparent) and thus thicker. Rectangles cover a size range larger than hexagons, and their tendency to orient relatively to the grid differ dependent on the crystal size. One way to explain this is to assume that the hexagons are, in fact, thinner, creating overall morphologies different from those of the rectangles. If this is the case, the crystallization might initiate by forming a thin layer on the *a*/*b* plane first, and then extend along the *c* direction.

## Transitions from amorphous precipitants to crystals

In addition to the regularly shaped objects, a number of amorphous ones of varying sizes were observed (see Fig. 3d, e, and bottom of Fig. 3c). Most of these had sizes 5–500 nm, but some others were quite large, ~1000 nm (= ~1 μm). These appear to be non-crystalline precipitates of the protein pot0434017.

Some objects observed are intermediate between crystals and amorphous precipitates. Thus, in Fig. 3c, a hexagonal crystal appears to be being formed from an amorphous precipitant. In Fig. 3a, a large hexagonal crystal appears to be growing by absorbing smaller amorphous precipitants surrounding. The fundamental form of this protein is a dimer. It is likely that aggregates of dimers are assembled into cylinders upon such transitions. Alternatively, disks composed of four dimers might be being transformed into helical turns of cylinders. 4)

## Formation of films by pot0434017

**Fig. 4:**
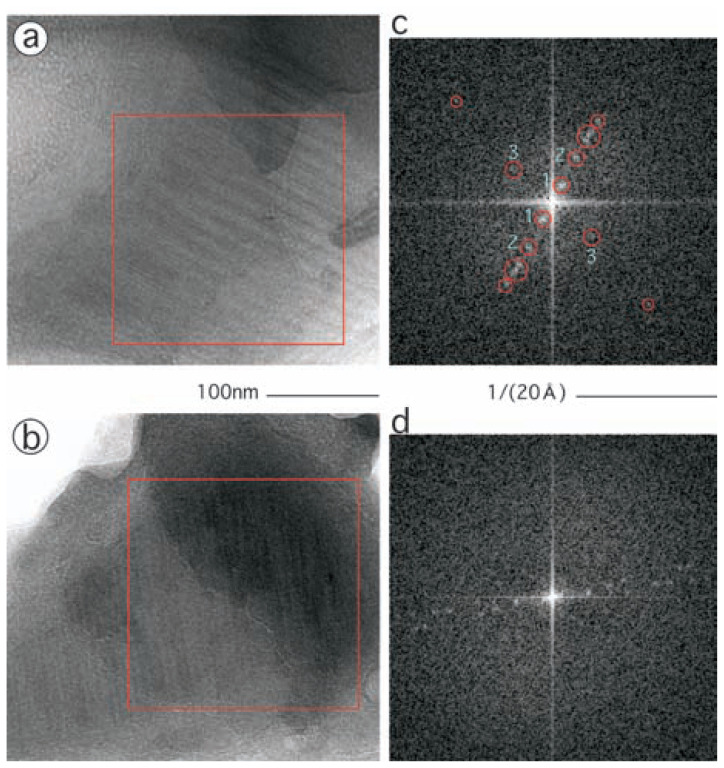
Assemblies of tubes in films of pot0434017 (a, b), and Fourier power spectra of parts of the images (c, d). The images (a) and (b) were recorded at the magnification of 55,000, and shown at the same scale. (c) is a Fourier power spectrum calculated from the region boxed in (a), and (d) of (c). In (c) some notable spots are circled and labeled.

In images of two pieces of films (Fig. 4a, b), tubes are observed running parallel to each other. The periodicity of the lateral arrangement of these tubes in Fig. 4a is ~150 Å. Each tube in Fig. 4a, in fact, can be interpreted as a projection of a hollow cylinder, since inside each two dense strips are separated by a gap of ~45 Å. These numbers have been confirmed by calculating a Fourier power spectrum (Fig. 4c): pairs of spots were identified along the equator (labeled 1 for the ~150 Å and 2 for the ~45 Å in Fig. 4b). In addition, another pair are positioned off-meridian (labeled 3 in Fig. 4c), indicating the presence of a component of a helical periodicity ~50 Å.

All these suggest that the tubes are the projections of helical cylinders assembled by pot0434017. The ~150 Å periodicity reflects the *a*/*b* unit length, 130.9 Å (Fig. 1a). Another equatorial ~45 Å periodicity reflects the separation between pairs of side-walls of the multimeric core formed by C-domians. The helical component, ~50 Å, corresponds to the helical pitch of the cylinder 47.4 Å.

Importantly the film appear to be a mono-layer of the cylinders assembled on the *a*/*c* or *b*/*c* planes (indicated by an arrow in Fig. 1a). Otherwise, in the power spectrum shown in Fig. 4c, the ~150 Å periodicity would not be identified. Instead, a series of spots could have been formed starting not from the observed ~150 Å but with half this size ~75 Å; between tubes observed in Fig. 4a additional tubes could have been projected from the nearby layer (Fig. 1a).

Since the amorphous ice formed on the grid has a depth, such a mono-layer is not necessarily be oriented parallel to the grid, but can be tilted around a pair of axes. In addition, the layer can have distortions, e.g. waving. The one shown in Fig. 4a appears to be rather flat and parallel to the grid, since the Fourier spots can be interpreted as already explained. However, another film shown in Fig. 4b appears to be tilted by ~40° around its *c* axis: the Fourier spots are found more outwards along the equator by ~30% (Fig. 4d). In fact, in Fig. 4c, the film is folded at the left upper part, and overlaid back on top of the original layer. The Fourier power spectrum (Fig. 4d) shows that the two layers are relative rotated by ~5°.
